# A Meta-Analysis of 13 Randomized Trials on Traditional Chinese Medicine as Adjunctive Therapy for COVID-19: Novel Insights into Lianhua Qingwen

**DOI:** 10.1155/2022/4133610

**Published:** 2022-10-30

**Authors:** Yi Lei, Huizhen Guan, Wenqiang Xin, Bi-Cheng Yang

**Affiliations:** ^1^Jiangxi Provincial Key Laboratory of Birth Defect for Prevention and Control, Jiangxi Provincial Maternal and Child Health Hospital, Nanchang 330006, China; ^2^School of Public Health, Nanchang University, Nanchang, China; ^3^Department of Neurosurgery, Tianjin Medical University General Hospital, Tianjin, China

## Abstract

The efficacy and safety of traditional Chinese medicine (TCM) paired with western medicine in the treatment of patients with COVID-19 remains controversial. This meta-analysis was performed to identify the effects of TCM. Seven electronic databases were reviewed from the inception of these databases to 30 June 2022. A quality assessment of the included studies was performed with the Cochrane Collaboration's tool to provide a score of high, unclear, or low risk of bias. The standard software program (Stata, version 12.0, statistical software) was used for endpoint analyses. A total of 13 RCTs involving 1398 patients conducted in China were included. The cross-sectional data from various studies were plotted, and the results illustrated that the statistically higher rates of total effectiveness (RR, 1.357; 95% CI, 1.259 to 1.464; *P* < 0.001), improvement of chest CT (RR, 1.249; 95% CI, 1.143 to 1.356; *P* < 0.001), and cough improvement (RR, 1.228; 95% CI, 1.057 to 1.570; *P* = 0.012) and a lower incidence of conversion to severe cases (RR, 0.408; 95% CI, 0.275 to 0.605; *P* < 0.001) were demonstrated in the TCM group than that of the control group. Of note, the subgroup on specific TCM of Lianhua Qingwen (LQ) revealed that the experiment group was associated with a higher rate of total effectiveness (RR, 1.248; 95% CI, 1.136 to 1.371; *P* < 0.001) and improvement of chest CT (RR, 1.226; 95% CI, 1.110 to 1.356; *P* < 0.001) and a lower rate of conversion to severe cases (RR, 0.469; 95% CI, 0.311 to 0.707; *P* < 0.001). However, there was no significant difference in fever improvement (RD, 0.110; 95% CI, -0.063 to 0.283; *P* = 0.213). The findings of this meta-analysis suggest that TCM combined with western medicine is more effective in treating COVID-19 via relieving symptoms, promoting patients' recovery, and cutting the rate of patients developing into severe conditions. However, given the relevant possible biases in our study, adequately powered and better-designed studies with long-term follow-up are required to reach a firmer conclusion.

## 1. Introduction

Despite the coronavirus disease 2019 (COVID-19) pandemic being first declared over two years, the COVID-19 pandemic has caused millions of deaths and significant changes in how people live [1]. A series of measures (such as face-masking, social distancing, and lockdowns) treatments have been widely developed worldwide to protect the population from severe disease and death during this time. Even though vaccinations are applicable primarily as a prophylactic intervention and the treatment of specific antiviral drugs is recommended, the increasing risks of toxic side effects and drug-resistant strains exist [2–6]. The focus primarily remains to aim at the acute phase of the disease via the mainstream strategies of symptomatic and supportive treatments. Given that the “best” medication for severe COVID-19 infection remains unknown, effective treatment of COVID-19 is urgently required. It is striking to note that traditional Chinese medicine (TCM), such as herbal medicine and acupuncture, has been a critical player in reversing the pandemic in China, which is an ancient medical/healing system. TCM developed and matured at least 2000 years ago through particular diagnostic approaches and a mixture of Chinese botanical drugs prescribed by Chinese herbalists for pattern identification to treat disease [7, 8]. Instead of aiming at killing pathogenic factors by allopathy, or excising the lesions of tissue via surgery, TCM focused on adjusting the balance of the body through regulating the microenvironment of the tissue and cells and even boosting the immunity of the body overall, suggesting these characters are superior advantages in the current situation of COVID-19 pandemic as an adjunctive therapy [7, 8]. A shred of emerging evidence has demonstrated that a great number of TMC providers combined with western medicine are associated with improving clinical symptoms in patients with COVID-19 [9, 10]. Contrary to extensive TCM herbal medicine practice in fighting COVID19, the published TCM clinical studies in COVID19 treatment were based on a small sample. It is urgent to integrate to provide more convincing data. As such, the present study is aimed at integrating the existing evidence to evaluate the efficacy and safety of TCM paired with western medicine in the treatment of patients with COVID-19 so that we could inform clinical practice. Moreover, a notion will be given to the efficacy and safety of a specific TCM, namely, Lianhua Qingwen (LQ), by performing a subgroup analysis.

## 2. Materials and Methods

This study adopted the Preferred Reporting Items for Systematic Reviews and Meta-Analyses statement as guidelines [11].

### 2.1. Literature and Search Strategy

A comprehensive literature search of several electronic databases, namely, Chinese Science and Technology Periodical Database, Wanfang database, PubMed, Cochrane Library, EMBASE, Web of Science (WOS), and China National Knowledge Infrastructure (CNKI), was performed by two researchers (Wenqiang Xin and Yi Lei) independently, from the inception of these databases to 30 June 2022. We retrieved studies assessing TCM in treating COVID-19 adopting the following: keywords and MeSH terms in domains of COVID-19, traditional Chinese medicine, and official Chinese terms for the 7 medicines (e.g., “Lianhua Qingwen” and “Qingfei Paidu Tang”). In addition, references from the identified reports, such as original studies, review studies, systematic reviews, and meta-analyses, were manually searched to identify other potential qualifying trials that this electronic search had not found.

### 2.2. Inclusion and Exclusion Criteria

If the trial met the following criteria in accordance with PICOS (participants, interventions, comparators, outcomes, and study design), the article was considered to be involved in this meta-analysis: (I) population: the study had limited comparison to the patients with COVID-19; (II) intervention: the study had strictly used TCM; (III) comparison: the study had compared the efficacy of TCM for COVID-19; (IV) outcome measures: one or more of the following outcomes were reported: total effective rate, improvement rate of chest CT, fever improvement rate, cough improvement rate, and ate of conversion to severe cases; and (V) study design: the study was an official, reported, and full-text randomized controlled trial (RCT).

The exclusion criteria were as follows: (I) in vitro and animal experiments, conference articles, commentary articles, and letters to the editor; (II) observational studies, systematic reviews, meta-analyses, case reports, and case series; (III) unclear patient characteristics and outcomes data; and (IV) studies of other types of viral pneumonia.

### 2.3. Data Extraction and Outcome Measures

Two researchers (Wenqiang Xin and Yi Lei) independently reviewed abstracts and full text, extracted data from all included studies, and discussed inconsistencies until consensus was obtained. The following important preplanned data elements were captured, including the basic data (first author name author, publication year, and patient age), study characteristics (sample size and follow-up period), and outcomes (total effective rate, the improvement rate of chest CT, fever improvement rate, cough improvement rate, and rate of conversion to severe cases). The primary endpoint was a composite of the total effective rate and improvement rate of chest CT. The secondary outcome measurements were those relevant to improving clinical symptoms, including fever improvement rate, cough improvement rate, and rate of conversion to severe cases.

### 2.4. Statistical Analysis

This study used a standard software program (Stata, version 12.0, statistical software (StataCorp LP, College Station, Texas, USA)) for endpoint analyses. When *I*^2^ > 50%, the data were deemed to have apparent heterogeneity. We conducted a meta-analysis using a random-effect model according to the Cochrane Handbook for Systematic Reviews of Interventions (version 5.1.0). Heterogeneity across trials was identified where each outcome uses *I*^2^ statistics (with *I*^2^ < 50% being low and *I*^2^ > 50% being apparent heterogeneity) and Cochran's *Q* (with *P* < 0.1 indicating significance). Otherwise, a fixed-effect model was adopted. Among all various discontinuous outcomes, rate difference (RD) or risk ratios (RRs) with 95% CIs were applied for the assessment.

## 3. Results

### 3.1. Search Results

**Figure 1:**
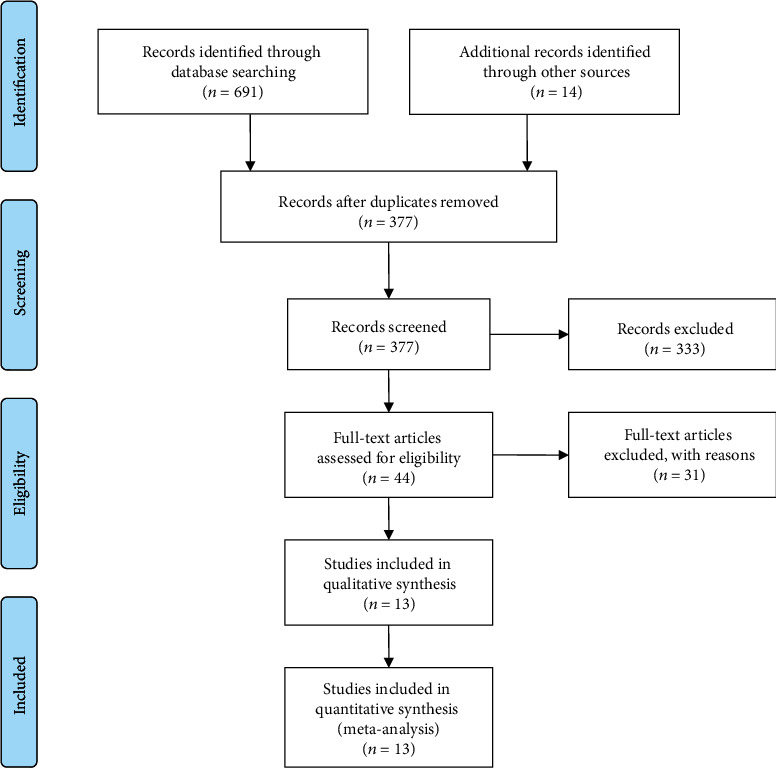
Flowchart of the study selection process.

Based on the proposed retrieval strategy and method, 691 reports were initially retrieved. Three hundred and twenty-two duplicated reports were removed. Three hundred thirty-three reports were further deleted by reviewing titles and abstracts; the full text of 44 papers was screened, and the 31 nonconforming papers were deleted according to the inclusion criteria, exclusion criteria, and data integrity. Finally, 13 [12–24] RCTs were involved, two of which are in English and the rest in Chinese. The specific screening process is shown in Figure 1.

### 3.2. Characteristics of Included Studies

A total of 13 RCTs [12–24] involving 1398 patients conducted in China were included, and among these studies, 11 were published in Chinese [12, 14–18, 20–24] and 2 were in English [13, 16]. Among these studies, 5 [12–16] of them adopted LQ treatment (capsules (0.35 g/capsule) and granules (6 g/bag)) in combination with western medicine therapy, including interferon-*α*, lopinavir/ritonavir, arbidol, and other antivirals). Two studies used 100 mL Qingfei Paidu decoction (bid) as adjunctive therapy for 10 days [20, 21]. Similarly, 2 studies adopted Toujie Quwen granules as adjunctive therapy [17, 18]. In addition, there are four articles, respectively, using Shufeng Jiedu capsules [19], Reyanning [22], Jinhua Qinggan granules [23], and Qingfei Touxie Fuzhengfang [24]. The duration of treatment varied from 5 to 15 days. More details are shown in Table 1.

### 3.3. Quality Assessment

Two independent reviewers (Wenqiang Xin and Yi Lei) have appraised the risk of bias in each of the detailed studies using the Cochrane Collaboration tool to provide a score of high, unclear, or low risk of bias. When differences arose in the process, they would get together and discuss them. Specifically, each included study was assessed for seven items: random sequence generation, allocation concealment, blinding of participants and personnel, blinding of outcome assessment, incomplete outcome data, selective reporting, and other sources of bias. Most of them showed a low risk of bias for random sequence generation, blinding of outcome assessment, incomplete outcome data, and selective reporting. Most of the included RCTs were of moderate quality, as provided in Table 2.

### 3.4. The Outcome of the Meta-Analysis

A total of 13 RCTs were eligible for analysis, with 1398 patients. The detailed results are shown in Table 3 and listed as follows.

### 3.5. Primary Endpoints

#### 3.5.1. Total Effective

**Figure 2:**
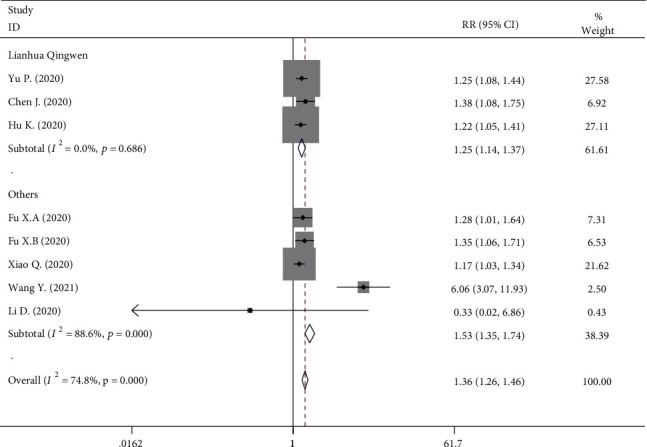
Forest plot on the assessment of the total effective rate.

Eight studies provided data on total effectiveness. In the TCM group (569 participants), the incidence of total effectiveness was 85.06%. In the control group (549 participants), the incidence of total effectiveness was 63.21%, with a statistically significant difference (RR, 1.357; 95% CI, 1.259 to 1.464; *P* < 0.001, Figure 2). However, significant heterogeneity among the studies was identified (*I*^2^ = 74.8%, *P* < 0.001). A subgroup was performed on a specific TCM of LQ. Similarly, the experiment group was associated with higher total effectiveness (RR, 1.248; 95% CI, 1.136 to 1.371; *P* < 0.001, Figure 2). The studies identified no significant heterogeneity (*I*^2^ = 0%, *P* = 0.686).

#### 3.5.2. Improvement of Chest CT

**Figure 3:**
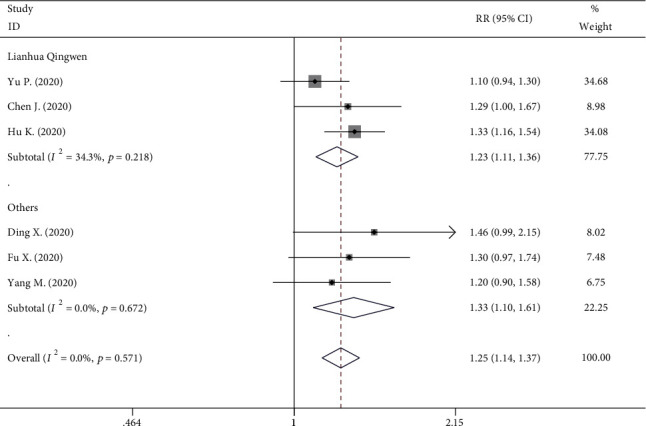
Forest plot on the assessment of the improvement rate of chest CT.

Six studies reported the number of patients with improvement in chest CT. The results showed 431 cases in the experiment group and 433 cases in the control group. No significant heterogeneity was identified (*I*^2^ = 0%, *P* = 0.571), and a fixed-effect model was conducted. The cross-sectional data from various studies were plotted and showed that the rate of improvement of chest CT was statistically higher in the TCM group (333/431) than that of the control group (268/433) (RR, 1.249; 95% CI, 1.143 to 1.356; *P* < 0.001, Figure 3). Similarly, a subgroup analysis on LQ indicated that it had a higher incidence of improvement of chest CT (RR, 1.226; 95% CI, 1.110 to 1.356; *P* < 0.001, Figure 3) with no significant heterogeneity (*I*^2^ = 34.3%, *P* = 0.218).

### 3.6. Second Endpoints

#### 3.6.1. Fever Improvement

**Figure 4:**
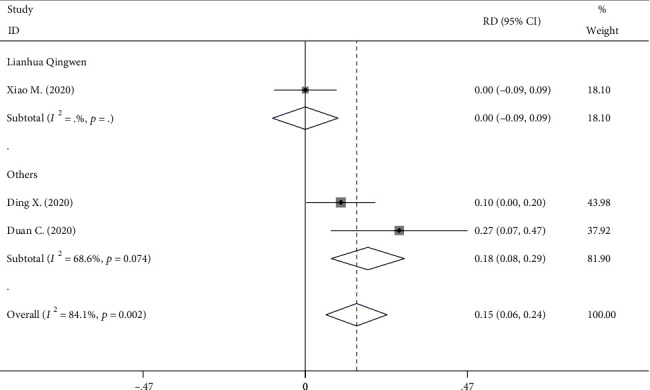
Forest plot on the assessment of the fever improvement rate.

Three RCT studies (141 and 99 patients in the TCM and control group, respectively) reported on fever improvement rate. Significant heterogeneity was observed, and a random-effect model was used (*I*^2^ = 84.1%, *P* = 0.002). The fever improvement rate between the experimental and control groups was not statistically significant (TCM: 127/141 vs. control: 78/99 RD, 0.110; 95% CI, -0.063 to 0.283; *P* = 0.213, Figure 4).

#### 3.6.2. Cough Improvement

**Figure 5:**
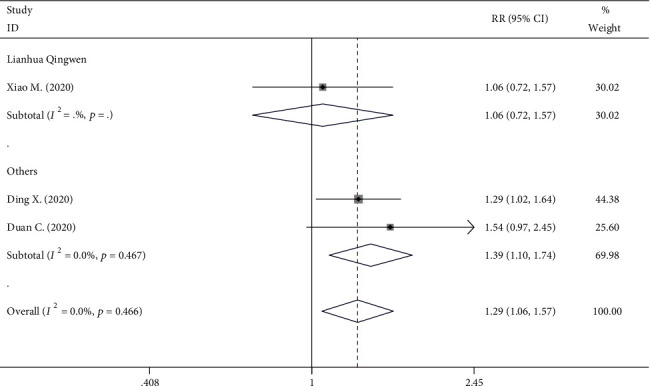
Forest plot on the assessment of the cough improvement rate.

Data regarding the information on cough improvement rate in COVID-19 patients treated with TCM were also available in three RCT studies (240 patients). The rate of cough improvement in the TCM group (99/137) showed a significantly higher tendency than the control group (60/103) (RR, 1.228; 95% CI, 1.057 to 1.570; *P* = 0.012, Figure 5), with significant heterogeneity (*I*^2^ = 0%, *P* = 0.466).

#### 3.6.3. Conversion to Severe Cases

**Figure 6:**
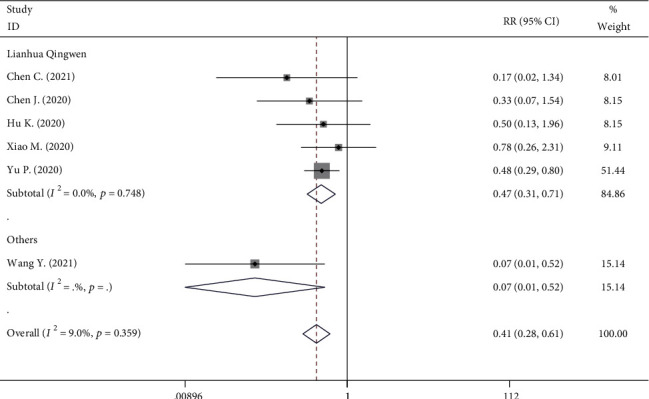
Forest plot on the assessment of the rate of conversion to severe cases.

Five RCTs provided the numbers of conversion to severe cases. A low heterogeneity was found (*I*^2^ = 9%, *P* = 0.187), and we used a fix-effect model. Among these studies, the incidence of conversion to severe cases in the TCM group is 6.25% (30 of 480), which is smaller than the non-TCM group (15.65%, 72 of 460). This comparison fully indicates that TCM was associated with a lower value of the rate of conversion to severe cases than placebo or nothing in COVID-19 patients (RR, 0.408; 95% CI, 0.275 to 0.605; *P* < 0.001, Figure 6). Similarly, a subgroup analysis on LQ indicated that it had a lower incidence of conversion to severe cases (RR, 0.469; 95% CI, 0.311 to 0.707; *P* < 0.001, Figure 6) with no significant heterogeneity (*I*^2^ = 0%, *P* = 0.748).

## 4. Discussion

COVID-19, firstly detected in China in 2019, was declared a pandemic by the World Health Organization in 2020 [25]. Although the practical option of antiviral therapy and vaccination is currently under evaluation and development, the management of COVID-19 generally focuses on supportive therapy through preventing respiratory failure. TCM is an ancient treatment strategy with abundant clinical experience and effective prescriptions to control and treat infectious diseases in about 500 epidemics that occurred in China over 3000 years [9, 26]. COVID-19 belongs to the plague in TCM with the etiology of epidemic factor exposure [27]. This comprehensive meta-analysis was based on 13 RCTs involving 1398 patients assigned to available TCM combined with western treatment or western treatment alone to compare the effects. The results demonstrated that for COVID-19 patients, TCM combined with western medicine treatment was more efficacious with total effectiveness, improvement of chest CT, and cough improvement than western medicine treatment alone. In addition, TCM was associated with a lower incidence of conversion to severe cases. Among these 13 RCTs, LQ occupied 5, with a bigger sample than any other Chinese herbal medicine products/decoction; therefore, it was picked up and did subgroup analysis. LQ was associated with a similar outcome of total effectiveness, improvement of chest CT, and conversion to severe cases. Given the lack of effective treatment, this study suggested TCM as adjunctive effective therapy for relieving the clinical symptoms of COVID-19.

The efficacy of various TCM on patients with COVID-19 has been previously confirmed by scientific studies. For example, Ding et al. [24] indicated that the treatment of COVID-19 with Qingfei Touxie Fuzhengfang was effective. Symptoms of COVID-19 patients could be alleviated by early and timely application of the combined solution of Toujie Quwen granules and Arbidol [18]. Li and Zhang [21] reported that Qingfei Paidu Tang could significantly improve the clinical symptoms of patients with severe COVID-19 and reduce the duration of hospital stay, and the therapeutic regimen is safe and reliable. Mechanically, the expression of T cell counts is controlled, and immune function is restored [11]. Besides that, the herbal drug components, such as ephedra and Poria, are one of the primary ingredients in most of the TCM medicinal formulae and contain the anti-inflammatory polysaccharides that play a vital role in repressing the cytokine storm [28, 29]. Therefore, the possible mechanism lies in the upregulation of “antiviral” factors and the downregulation of “proinflammatory” factors.

Of note, the LQ capsule, one of the most studied TCM, a patented product that has been marketed for the severe acute respiratory syndrome epidemic since the outbreak of severe acute respiratory syndrome in 2003 in China, can significantly boost the improvement and decrease the duration of fever, fatigue, and coughing [13]. In this study, we searched a total of 8 studies evaluating the effect of LQ in COVID-19, five of which were RCTs [12–16] and the rest were in non-RCTs [30–32]. This study demonstrated that the LQ was associated with a higher rate of total effectiveness (*P* < 0.001) and improvement of chest CT (*P* < 0.001) and a lower rate of conversion to severe cases (*P* < 0.001). Several studies have revealed that LQ has the potential to inhibit the release of tumor necrosis factor-*α* (TNF-*α*), interleukin-6 (IL-6), macrophage chemokine protein-1 (MCP-1), and inducible protein 10, suggesting that it can alleviate the lung injury partly via inflammatory cell infiltration [33]. Moreover, the evidence revealed that the LQ capsule could suppress the cytopathic effect of the virus in vitro, decrease the viral loads in the cytoplasm and cellular, and inhibit virus replication [33, 34].

Compared with many other antiviral drugs, the adverse effects of LQ are relatively mild. Data from the National Adverse Drug Reaction Monitoring and Direct Reporting System of China (Adverse Reaction Supervision System) show that the incidence is about 1 in 100,000 [35], which is a rare grade in the treatment of COVID-19. Common adverse reactions in the previous clinical use of LQ were mainly focused on gastrointestinal reactions [36], and adverse effects on the liver were relatively rare. Additionally, most drug-related liver damage is generally self-limiting and can be relieved after discontinuing the causative drug [37]. However, adequate attention should still be paid to the adverse drug reactions during treatment, assessing patients' liver function levels and paying attention to monitoring patients' transaminases and bilirubin levels, especially in patients with combined underlying liver disease, to achieve reasonable safety and effectiveness of clinical drug use.

The following limitations of this study should be noted. The present study only compares clinical outcomes relevant to the safety and efficiency of TCM therapy due to relatively little data on these outcomes with the same long-term follow-up period. The duration and dosage of TCM were not standardized, which may confound the outcomes. Besides that, the prominent inclusion and exclusion criteria of the included RCTs and the participants' characteristics were varied, potentially resulting in bias. Some of the included RCT studies were open-label (non-double-blind or single-blind) designs, which may have influenced the conduct and outcomes of the studies, such as patient dropout, investigator assessment of overall effectiveness and conversion to remission, and assessment of chest CT. However, the treatment group was not known to the investigators conducting the trial, which is not considered to have influenced the overall conclusions. Finally, although all included studies noted an excellent effect of TCM combination for new crowns, the effect may have been overestimated due to the lack of allocation concealment or blinding of the treatment group.

## 5. Conclusion

The findings of this meta-analysis suggest that TCM combined with western medicine is more effective in treating COVID-19 via relieving symptoms, promoting patients' recovery, and cutting the rate of patients developing into severe conditions. However, given the relevant possible biases in our study, adequately powered and better-designed studies with long-term follow-up are required to reach a firmer conclusion.

## Figures and Tables

**Table 1:** The main characteristics of the randomized controlled trials are included in the meta-analysis.

Author	Year	Country	Traditional Chinese	Sample	Patient average age (y)		TCM	Treatment	Ref.
			Medicine (TMC)	Size	Control	Experiment	Dosage	Duration	
Chen et al.	2021	China	Lianhua Qingwen capsules	60	49.5 ± 5.1	50.2 ± 5.1	4 capsules, bid	15 days	[12]
Hu et al.	2020	China	Lianhua Qingwen capsules	284	51.8 ± 14.8	50.4 ± 15.2	6 g, tid	7 days	[13]
Yu	2020	China	Lianhua Qingwen capsules	295	47.3 ± 8.7	48.3 ± 9.6	4 capsules, tid	Not reported	[14]
Chen et al.	2020	China	Lianhua Qingwen capsules	70	45.2 ± 4.7	44.8 ± 4.9	4 capsules, tid	14 days	[15]
Xiao et al.	2020	China	Lianhua Qingwen capsules	121	53.9 ± 13.9	52.7 ± 14.0	6 g, tid	14 days	[16]
Fu et al.	2020	China	Toujie Quwen granules	36	43.68 ± 6.45	43.26 ± 7.15	Not reported, bid	10 days	[17]
Fu et al.	2020	China	Toujie Quwen granules	38	44.68 ± 7.45	45.26 ± 7.25	Not reported, bid	15 days	[18]
Xiao et al.	2020	China	Shufeng Jiedu capsules	200	60.90 ± 8.70	60.90 ± 8.70	2.08 g, tid	14 days	[19]
Wang et al.	2021	China	Qingfei Paidu decoction	71	49.4 ± 13.3	48 ± 13.2	100 mL, bid	10 days	[20]
Li and Zhang	2020	China	Qingfei Paidu decoction	12	50.00 ± 10.00	52.00 ± 6.56	100 mL, bid	Not reported	[21]
Yang et al.	2020	China	Reyanning	49	47.17 ± 16.57	50.35 ± 13.37	Not reported	Not reported	[22]
Duan et al.	2020	China	Jinhua Qinggan granules	62	50.29 ± 13.17	51.99 ± 13.88	10 g, tid	5 days	[23]
Ding et al.	2020	China	Qingfei Touxie Fuzhengfang	100	50.8 ± 23.5	54.7 ± 21.3	150 mL, bid	10 days	[24]

**Table 2:** Cochrane Collaboration's tool for quality assessment in all included trials.

Trials	Year	Sequence generation	Allocation concealment	Blinding of outcome assessors	Incomplete outcome data	Selective outcome reporting	Others
Chen et al.	2021	Low	Unclear	Low	Low	Low	Low
Hu et al.	2020	Low	Low	Low	Low	Low	Low
Yu	2020	Low	Low	Unclear	Low	Low	Unclear
Chen et al.	2020	Low	Low	Low	Low	Low	Unclear
Xiao et al.	2020	Unclear	High	Unclear	Low	Low	Low
Fu et al.	2020	Low	Low	Low	Low	Low	Low
Fu et al.	2020	Low	Low	Unclear	Low	Low	Unclear
Xiao et al.	2020	Unclear	Low	Low	Low	Low	Unclear
Wang et al.	2021	Low	Low	Low	Low	Low	Unclear
Li and Zhang	2020	Low	Low	Unclear	Low	Low	Low
Yang et al.	2020	Low	Unclear	Low	Low	Low	Unclear
Duan et al.	2020	Low	Low	Low	Low	Low	Unclear
Ding et al.	2020	Low	Low	Low	Low	Low	Unclear

**Table 3:** The outcomes of this meta-analysis.

Outcomes	Studies Numbers	Sample size		Overall effect			Heterogeneity	
		Experiment	Control	Effect estimates	95% CIs	*P* value	*I* ^2^ (%)	*P* value
*Traditional Chinese medicine as the adjunctive therapy for COVID-19*								
Total effective	8	484/569	347/549	RR = 1.357	1.259-1.464	*P* < 0.001	74.8%	*P* < 0.001
Improvement of chest CT	6	333/431	268/433	RR = 1.249	1.143-1.356	*P* < 0.001	0.0%	*P* = 0.571
Fever improvement	3	127/141	78/99	RD = 0.110	-0.063-0.283	*P* = 0.213	84.1%	*P* = 0.002
Cough improvement	3	99/137	60/103	RR = 1.288	1.057-1.570	*P* = 0.012	0.0%	*P* = 0.466
Conversion to severe cases	5	30 of 480	72 of 460	RR = 0.408	0.275-0.605	*P* < 0.001	9%	*P* = 0.359
*The subgroup of Lianhua Qingwen as the adjunctive therapy for COVID-19*								
Total effective	3	264/324	216/331	RR = 1.248	1.136-1.371	*P* < 0.001	0.0%	*P* = 0.686
Improvement of chest CT	3	252/324	210/331	RR = 1.226	1.110-1.356	*P* < 0.001	34.3%	*P* = 0.218
Conversion to severe cases	4	29/410	63/417	RR = 0.469	0.311-0.707	*P* < 0.001	0.0%	*P* = 0.748

Note: CIs = confidence intervals; RD = rate difference; OR = odds ratio.

## Data Availability

The data that support the findings of this study are available from the corresponding author upon reasonable request.
